# Operative Management of a Perforated Duodenal Diverticulum With a Retroperitoneal Abscess

**DOI:** 10.7759/cureus.117793

**Published:** 2026-10-12

**Authors:** Mason A Johnson, Jessica E Echevarria, Brian Gibson, Samuel H Berding

**Affiliations:** 1 General Surgery, Alabama College of Osteopathic Medicine, Dothan, USA; 2 General Surgery, Northeast Georgia Medical Center Gainesville, Gainesville, USA; 3 Trauma and Acute Care Surgery, Northeast Georgia Medical Center Gainesville, Gainesville, USA

**Keywords:** acute abdomen, diverticulectomy, duodenal diverticulum, duodenal perforation, emergency surgery, exploratory laparotomy, gastrointestinal perforation, omental reinforcement, retroperitoneal abscess, second portion of duodenum

## Abstract

Duodenal diverticula are common and usually asymptomatic; however, perforation is a rare and potentially life-threatening complication. Perforation may produce nonspecific clinical and radiographic findings and can be difficult to distinguish from other causes of perforated viscus because much of the duodenum is retroperitoneal. We describe a 71-year-old male with cardiac amyloidosis, hypertension, atrial fibrillation on apixaban, and congestive heart failure who was transferred after more than one day of predominantly epigastric abdominal pain. Computed tomography demonstrated extraluminal air and retroperitoneal fluid in the region of the duodenum concerning for perforated viscus. Exploratory laparotomy revealed a perforated diverticulum arising from the lateral aspect of the second portion of the duodenum with a contained retroperitoneal abscess. The diverticulum was resected using a linear stapler, followed by staple-line imbrication and omental reinforcement. Histopathologic examination confirmed a duodenal diverticulum with marked surrounding acute serositis compatible with perforation. Postoperative upper gastrointestinal imaging demonstrated no contrast extravasation, and there was no clinical evidence of duodenal leak during the index hospitalization. This case highlights the diagnostic challenge of duodenal diverticular perforation and demonstrates that, when local anatomy and tissue quality permit, stapled diverticulectomy with reinforcement can provide definitive source control without more complex duodenal reconstruction.

## Introduction

Duodenal diverticula are the second most common intestinal diverticula after those of the colon and occur most frequently within the second portion of the duodenum (D2), often near the major duodenal papilla [1]. Most remain asymptomatic, although complications include inflammation, hemorrhage, obstruction, and perforation.

Perforation is uncommon and can be diagnostically challenging because the second and third portions of the duodenum are retroperitoneal. Thorson et al. identified 162 cases in the historical literature, while a subsequent systematic review identified 47 adequately documented cases published between 2008 and 2020 [2,3]. Treatment ranges from conservative management in carefully selected stable patients to operative intervention. When surgery is required, the approach is determined largely by tissue quality, diverticular anatomy, and the relationship of the lesion to the ampulla and pancreaticobiliary structures [2,3].

We present a perforated D2 duodenal diverticulum with a contained retroperitoneal abscess managed by stapled diverticulectomy, staple-line imbrication, and omental reinforcement.

## Case presentation

A 71-year-old male with a history of cardiac amyloidosis, hypertension, atrial fibrillation on apixaban, and congestive heart failure was transferred from an outside facility after one day of predominantly epigastric abdominal pain that progressively worsened with no previous history of similar symptoms. His prior surgical history was notable for laparoscopic inguinal hernia repair.

On arrival, the patient was hemodynamically stable and afebrile. Abdominal examination demonstrated epigastric tenderness with guarding. Initial laboratory evaluation demonstrated a white blood cell count of 8.6 K/µL with 11% band neutrophils, hemoglobin of 12.6 g/dL, and platelet count of 132 K/µL without significant electrolyte or renal abnormalities (Table 1).

**Table 1:** Patient laboratory values at presentation ^^^ indicates a value above the reference range; ^v^ indicates a value below the reference range

Parameter	Patient Value	Reference Range
WBC	8.6	4.8-10.8 x 10^3^ K/µL
Band neutrophils	11^^^	0-3%
Hemoglobin	12.6^v^	14.0-18.0 g/dL
Hematocrit	36.4^v^	42.0-52.0%
Platelets	132	130-400 K/µL
Sodium	139	135-148 mmol/L
Potassium	4.1	3.5-5.2 mmol/L
Chloride	105	100-110 mmol/L
CO₂	24	21-32 mmol/L
BUN	22	5-23 mg/dL
Creatinine	1.10	0.80-1.30 mg/dL
Glucose	149^^^	65-99 mg/dL

Computed tomography of the chest, abdomen, and pelvis with intravenous contrast demonstrated extraluminal air with retroperitoneal fluid in the region of the duodenum, concerning for a perforated viscus (Figure 1). The preoperative working diagnosis was perforated peptic ulcer.

**Figure 1:**
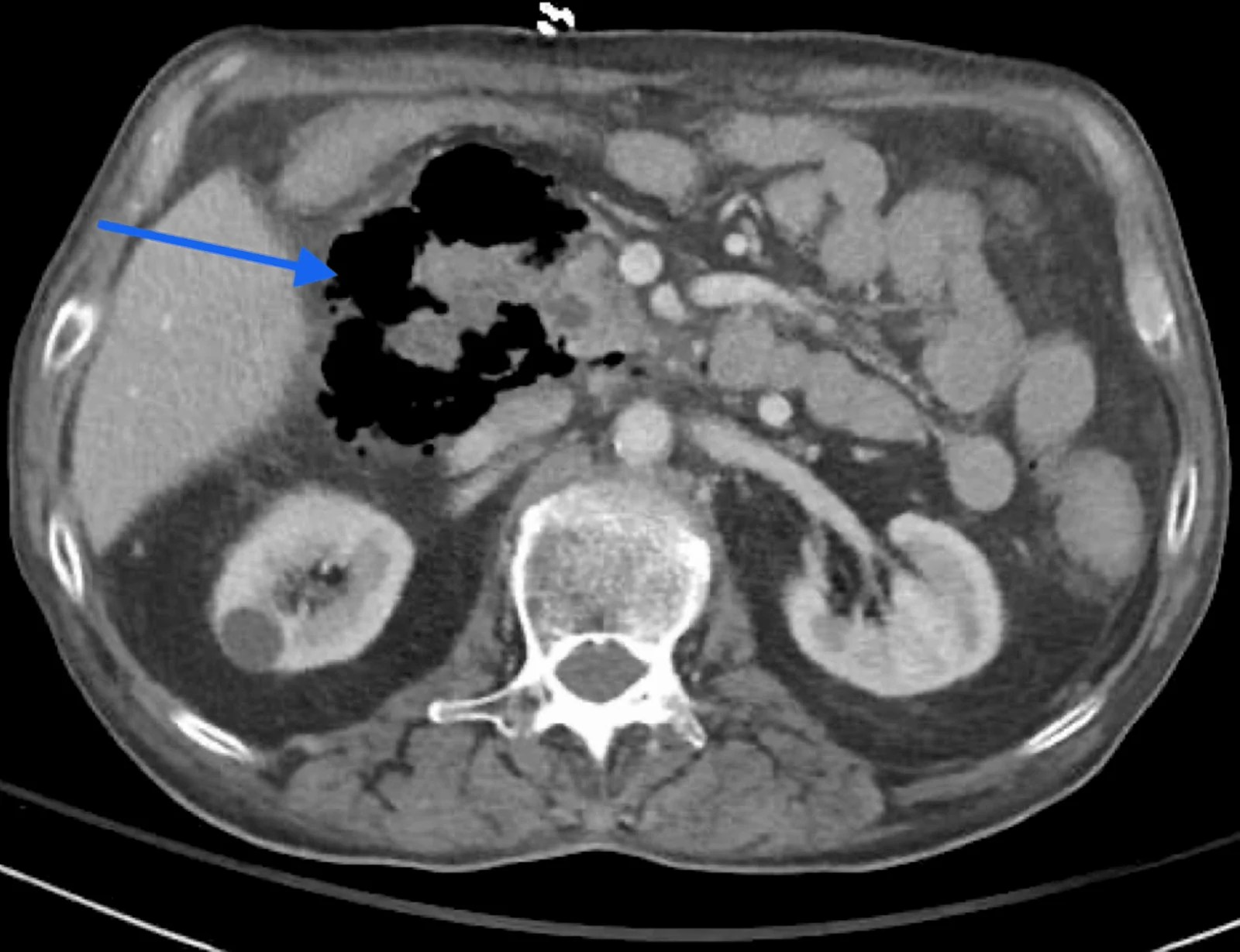
Computed tomography of the upper abdomen at initial presentation Axial contrast-enhanced computed tomography demonstrating extraluminal air adjacent to the second portion of the duodenum (blue arrow) with retroperitoneal extension, concerning for perforated viscus.

Because the patient had recently taken apixaban, anticoagulation was reversed with four-factor prothrombin complex concentrate. He was maintained nil per os, intravenous piperacillin-tazobactam was continued, and he was taken urgently for exploratory laparotomy.

Intervention

A midline laparotomy was performed. Exploration demonstrated edematous and matted omentum within the right upper quadrant. The omentum was mobilized, the hepatic flexure was freed, and a Kocher maneuver was performed to expose the duodenum. Enteric contamination was encountered, and a perforated diverticulum arising from the lateral (antimesenteric) aspect of D2 was identified with a contained abscess within the retroperitoneum (Figure 2).

**Figure 2:**
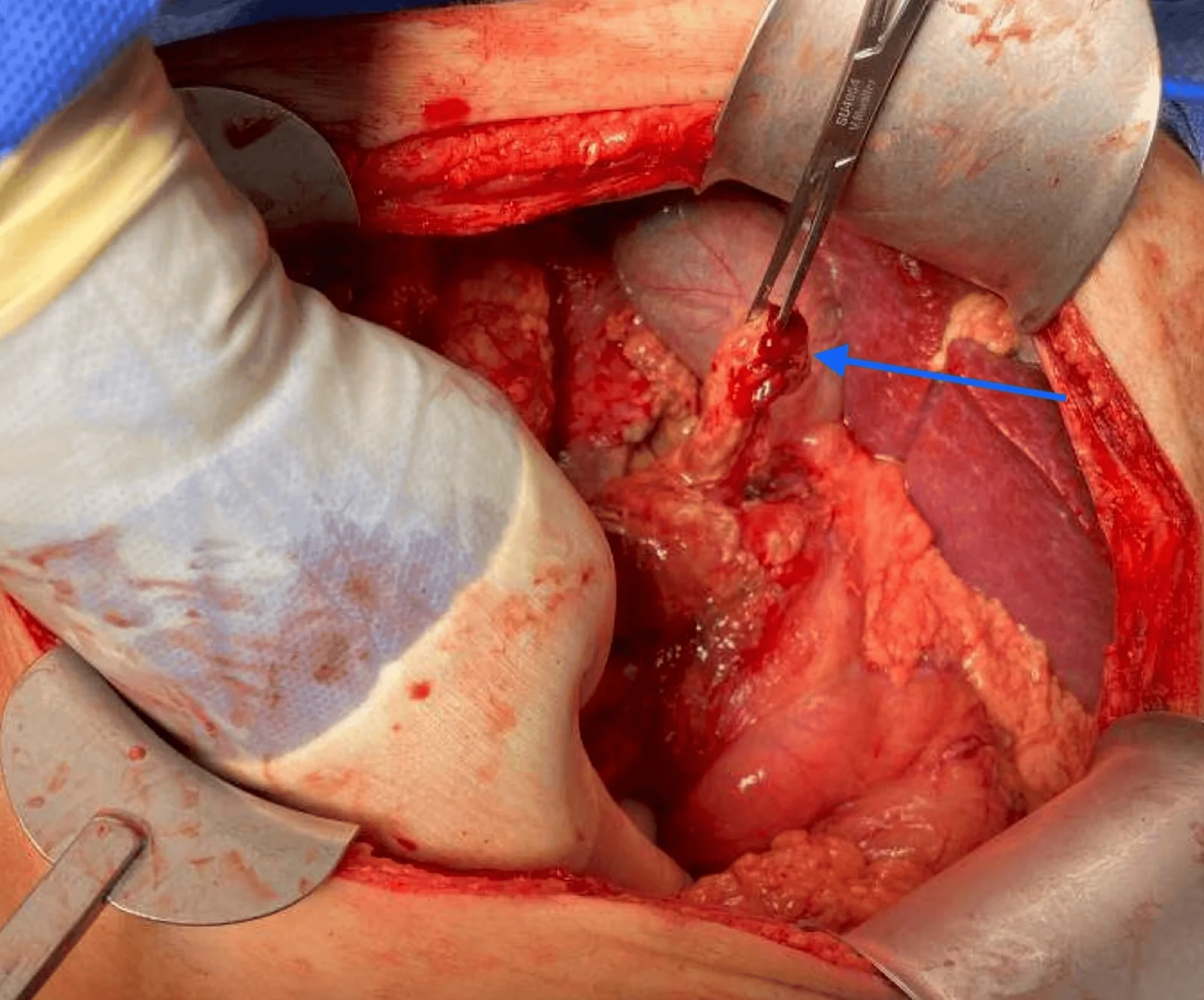
Perforated second portion duodenal diverticulum Intraoperative view following exposure and Kocherization of the duodenum demonstrating the perforated diverticulum arising from the lateral (antimesenteric) aspect of D2 (blue arrow). The tip of an Allis clamp identifies the site of perforation. The gallbladder and liver are visible posterior to the diverticulum.

The diverticulum was resected using a TA-60 blue-load linear stapler. The staple line was imbricated with 3-0 silk sutures. The right upper quadrant was thoroughly irrigated, hemostasis was achieved, and omentum was positioned over the reinforced staple line. A nasogastric tube was placed in the stomach, and a closed-suction drain was positioned adjacent to the repair. The patient tolerated the procedure without intraoperative complications and was transferred to the post-anesthesia care unit in stable condition.

Pathology

Histopathologic examination of the resected specimen confirmed a duodenal diverticulum with marked surrounding acute serositis compatible with perforation. The specimen measured 3.0 × 3.0 × 1.8 cm. Gross examination demonstrated roughened gray tan serosa with focal hemorrhage and flattened light tan mucosa. No luminal mass was identified. Histopathology slide images were not available because digital images were not obtained during routine pathologic evaluation.

Outcome

The patient underwent nasogastric decompression of his stomach during the early postoperative period. An upper gastrointestinal contrast study using Gastrografin on postoperative Day 1 demonstrated no evidence of extravasation from the duodenal repair. Oral intake was subsequently resumed and advanced as tolerated. There was no clinical evidence of duodenal leak during the index hospitalization, and drain output remained non-bilious.

At discharge on postoperative Day 9, the patient was tolerating a regular diet without nausea or vomiting and had adequate pain control. He was discharged home with the closed-suction drain in place.

Because the patient lived a significant distance from the treating facility, he elected to continue postoperative care with a surgeon closer to his residence. The drain was subsequently removed by that surgeon; however, additional long-term follow-up was unavailable.

## Discussion

Duodenal diverticular perforation is uncommon and most frequently involves the second or third portions of the duodenum. In the review by Thorson et al., 60 of 61 contemporary perforations occurred in D2 or D3 [2]. Reported etiologies include diverticulitis, enterolithiasis, ulceration, foreign bodies, trauma, and iatrogenic injury [2]. Histopathology in the present case confirmed a duodenal diverticulum with acute serositis compatible with perforation but did not identify a specific cause. The lesion is therefore best characterized as a perforated duodenal diverticulum rather than perforated diverticulitis.

Preoperative diagnosis may be difficult because retroperitoneal perforation can produce limited or nonspecific abdominal findings. Computed tomography may demonstrate duodenal wall thickening, surrounding inflammatory change, and adjacent retroperitoneal air or fluid [3,4]. Kapp et al. reported CT as the primary diagnostic modality in 43 of 47 patients, but the perforated diverticulum itself was prospectively identified in only 28 cases [3]. Similarly, imaging in our patient localized the suspected perforation to the duodenal region, while the definitive diagnosis was established during exploration.

Management is determined by clinical stability and the extent of perforation. Conservative treatment using bowel rest, gastric decompression, broad-spectrum antibiotics, nutritional support, and selective drainage has been successful in appropriately selected patients [3,5]. In the systematic review by Kapp et al., 16 of 47 patients were initially treated nonoperatively, although five ultimately required surgery [3]. Our patient's abdominal guarding and radiographic evidence of perforation prompted urgent exploration, allowing both definitive diagnosis and source control.

When surgery is required, tissue quality, diverticular morphology, and proximity to the ampulla are central to operative planning. Diverticulectomy with stapled or hand-sewn closure is the most commonly reported procedure when local anatomy is favorable [3,6]. Rossetti et al. described diverticular resection with direct closure in the majority of patients in their seven-patient series [6]. Moysidis et al. reported stapled diverticulectomy with suture reinforcement, while McKinley et al. emphasized assessment of the diverticulum's relationship to the ampulla before stapled resection [7,8]. Bunjo et al. described imbrication with omental reinforcement when the relationship to the ampulla made diverticulectomy less favorable [9]. In the present case, the lateral (antimesenteric) D2 location permitted localized stapled diverticulectomy. Staple-line imbrication and omental reinforcement provided additional coverage without requiring diversion or more extensive reconstruction.

Postoperative duodenal leak remains an important concern following operative repair. Fistulas have been reported after both localized diverticulectomy and more extensive reconstruction [3,6]. In our patient, postoperative contrast imaging demonstrated no extravasation, and drain output remained non-bilious through discharge.

This report is limited by the absence of long-term follow-up after the patient transitioned postoperative care to a surgeon closer to his residence.

## Conclusions

Perforated duodenal diverticula are uncommon and may be difficult to diagnose because of their retroperitoneal location. Imaging localized a suspected duodenal region perforation, while exploratory laparotomy established the definitive diagnosis of a perforated D2 diverticulum with a contained retroperitoneal abscess. Favorable local anatomy permitted stapled diverticulectomy with staple-line imbrication and omental reinforcement, with no evidence of postoperative duodenal leak during the index hospitalization. Recognition of this uncommon diagnosis and careful assessment of local D2 anatomy are important in selecting an operative approach that provides source control while minimizing the need for more extensive reconstruction.
